# Applied Anatomy

**Published:** 1905-09

**Authors:** 


**Applied Anatomy.** By EDWARD H. TAYLOR, M.D. Pp. xxvii., 738. London: Charles Griffin and Company Limited. 1904.

This is the most complete work of the kind which we have seen. It contains over 700 pages. It treats the subject carefully, methodically, and fully from beginning to end. Not only is it interesting and useful to the student working for his finals, but it is a book we feel sure which will be used by surgeons as a work of reference.

While the student is still in the dissecting-room it is generally a mistake for him to become too enamoured of surgery, and especially does it waste his time to make a practice of going to operations; but there is no doubt that this book will appeal to him not only from the interest it adds to his anatomical work, but because it will be read as a relaxation from what is sometimes considered the monotony of anatomy. About the work itself, there is not much to say by way of description. It takes the parts in regions, runs over the main anatomical points, and then in a way which only an experienced surgeon could have done it points out the various injuries and diseases to which these parts are liable, and the importance in each case of the knowledge of the anatomy in diagnosis and treatment. The illustrations are numerous, artistic and particularly clear. The book everywhere bears the stamp of long and patient work, and is one which is sure to quickly come into general request.

